# Genotypic characterization of *Orientia tsutsugamushi* from patients in two geographical locations in Sri Lanka

**DOI:** 10.1186/s12879-016-2165-z

**Published:** 2017-01-13

**Authors:** Ranjan Premaratna, Lucas S. Blanton, Dilhar N. Samaraweera, G. Nalika N. de Silva, Nilmini T. G. A. Chandrasena, David H. Walker, H. J. de Silva

**Affiliations:** 1 Department of Medicine, Faculty of Medicine, University of Kelaniya, Thalagolla Road, PO Box 6, Ragama, Sri Lanka; 2 Division of Infectious Diseases, Department of Internal Medicine, University of Texas Medical Branch, Galveston, TX USA; 3 Medical Unit, Base Hospital, Balapitiya, Sri Lanka; 4 Paediatric Unit, Base Hospital, Balapitiya, Sri Lanka; 5 Department of Parasitology, Faculty of Medicine, Universityof Kelaniya, Thalagolla Road, PO Box 6, Ragama, Sri Lanka; 6 Department of Pathology, University of Texas Medical Branch, Galveston, TX USA

**Keywords:** Orientia tsutsugamushi, Scrub typhus, Genotypes, Sri Lanka

## Abstract

**Background:**

To date more than 20 antigenically distinct strains of *Orientia tsutsugamushi* (OT) reported within the tsutsugamushi triangle that cause an undifferentiated acute febrile illness in humans. Genotypic characterization of OT in different geographic regions or within the same country, is important in order to establish effective diagnostics, clinical management and to develop effective vaccines. Genetic and antigenic characterization of OT causing human disease in OT-endemic regions is not known for Sri Lanka.

**Methods:**

Adult patients and children who were admitted with an acute febrile illness and presumed to having acute scrub typhus based on presence of an eschar and other supporting clinical features were recruited. Eschar biopsies and buffy coat samples collected from patients who were confirmed having OT by IFA were further studied by real time PCR (Orientia 47 kD) and nested PCR (Orientia 56 kD) amplification. DNA sequences were obtained for 56 kD gene amplicons and phylogenetic comparisons were analyzed using currently available data in GenBank [Neucleotide substitution per 100 residues, 1000 Bootstrap Trials].

**Results:**

Twenty eschar biopsies (Location1,19, Location 2,1) and eight buffy coat samples (Location1,6, Location2,2) examined by real time PCR revealed *Orientia* amplicons in 16 samples. DNA sequences were obtained for the 56 kD gene amplicons in 12 eschars and 4 buffy coat samples. The genotypes of the Location1 samples revealed that, 7 exhibiting close homology with JP1 [distantly related to UT177 Thai (Karp related)], five had close homology with Kato strain, two had close homology with JGv and JG AF [Distantly related to Kawasaki M63383] and one had close homology with Gilliam strain. The Location 2 strain was closely related to Kuroki-Boryong L04956, the genotype which is distributed in far eastern Asia. Similar to other patients in the cohort this patient also had never travelled out of Sri Lanka.

**Conclusions:**

We observed all three main OT genotypes in Sri Lanka, and the majority fell into Thai Karp related clade. These results demonstrate great antigenic diversity of OT in the studied areas of Sri Lanka.

**Electronic supplementary material:**

The online version of this article (doi:10.1186/s12879-016-2165-z) contains supplementary material, which is available to authorized users.

## Background

Scrub typhus (ST), or tsutsugamushi disease, is an acute febrile illness in humans caused by infection with *Orientia tsutsugamushi* (OT) following a bite of an infected mite vector of the genus *Leptotrombidium* [1]. Scrub typhus is endemic in the Asia-Pacific region, extending from Afghanistan to China, Korea, the islands of the western Pacific and Indian Oceans, and northern Australia [2, 3]. This endemic region is often referred to as the tsutsugamushi triangle, and hosts approximately 1 billion people [4]. The vectors can be found in a variety of ecological conditions, from the mountainous regions of northern India to the tropical climates of the Malay Peninsula and Indonesia [2]. Transovarial transmission of OT within vectors appears to be essential to maintenance of the agent in nature; thus, the mite serves as both the vector and the reservoir [2]. Transmission of the etiologic agent to vertebrate hosts occurs during feeding of the larval or “chigger” stage of mites [2]. While *Orientia* is vertically maintained in *Leptotrombidium* mite populations, it may be transmitted horizontally from mites to vertebrate hosts [2]. The transmission to humans is incidental. Currently, there is no vaccine against ST [5].

The disease mimics several other tropical febrile illnesses, and can vary from mild to fatal disease, with reported mortality rates of 35%–50% during the pre-antibiotic era [6, 7] to 7–9% currently, in different geographical regions [5]. Although detection of eschars assists early diagnosis, the occurrence of this sign is highly variable and depends on the host, geographical region, and possibly the bacterial strain [8]. ST became more familiar during World War II, when soldiers deployed to endemic regions were affected in great numbers [2]. Research since World War II has highlighted dramatic antigenic variation among strains of OT [2] Although great inter-strain variability in virulence has been shown in mouse models, it is not clear whether virulence for mice can be directly applicable to humans [2]. However, the antigenic variation may result in a spectrum of illness from very mild to disease that is often fatal when untreated [2]. To date, 20 antigenically distinct strains of OT have been reported, including the initially serologically distinguished prototypic strains Karp, Gilliam, and Kato [9]. The introduction of chloramphenicol and, later, the tetracyclines dramatically eliminated mortality among cases where treatment was started early [10–12]. Although relapses and reinfections occur [12], infection is generally responsive to treatment with antibiotics such as doxycycline and azithromycin, even when antigenically diverse strains are involved [2].

The documented history of scrub typhus in Sri Lanka dates back to the Second World War [5]. Similar to other countries in the tsutsugamushi triangle where scrub typhus is endemic, Sri Lanka also experiences a variable burden due to the illness. The incidence of the disease seems to be based on human activity, and ecological and climatic factors. Although OT in Sri Lanka is confirmed with IFA-based diagnostic techniques, the genotypic characterization of infecting OT strains is unknown. Such genotypic characterization of OT is important to establish effective diagnostics and to develop effective vaccines.

**Fig. 1:**
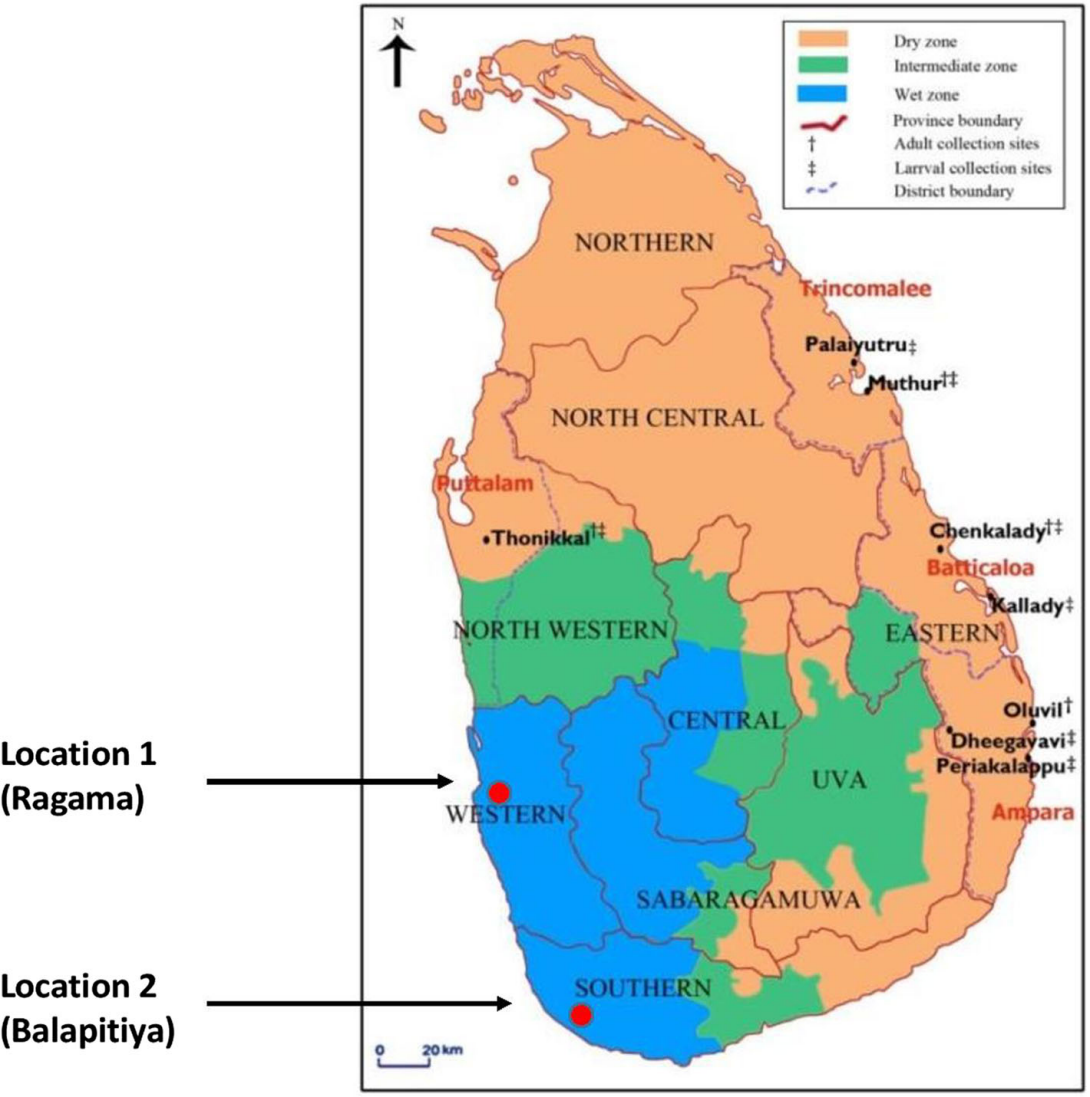
A map of Sri Lanka to highlight the geographical locations of the two study sites

The current study was designed to identify the strains of OT causing clinical illness in two areas along the western coastal belt of Sri Lanka. Both areas (Fig. 1), which are approximately 100 km apart, are endemic for OT. They are likely to have similar ecological characteristics, both are in the wet zone and 5–10 km from the coast.

## Methods

Paediatric and adult patients who presented with an acute febrile illness to Balapitiya Base Hospital (Location 1) and the Professorial Medical Unit of Colombo North Teaching Hospital (Location 2) in Sri Lanka were examined clinically in order to identify an eschar; the essential inclusion criteria for the study. Consecutive patients who fulfilled the essential criteria (acute fever and an eschar) were recruited for the study. For patients who were more than 18 years of age a punch biopsy of the eschar was performed after obtaining informed written consent. For those who were less than 18 years old, as they were considered not competent for consenting according to the Sri Lankan law, a 3 cc sample of blood was collected after obtaining informed written consent from their guardians. Patients who were considered severely ill, declined to consent, or had received anti-rickettsial antibiotics for more than 24 h were excluded from the study. Relevant demographic, clinical, and laboratory data of all patients were collected into a predesigned data collectionform.

Biopsy tissue and buffy coat samples were stored in RNA*later* (Life Technologies, Grand Island, NY) according to the manufacturer’s instructions. The samples were stored at−80°C until they were sent to the University of Texas Medical Branch, Galveston, USA, for further analysis. The patients were further tested by serology to detect IgG antibodies against Karp, Kato or Gilliam antigens by IFA in order to confirm OT infection. The IFA-IgG test was done using antigen coated slides procured from Fuller Laboratories (Fullerton, California, USA) adhering to manufacturer’s instructions.

Prior to processing, biopsy tissues stored in RNA*later* were rinsed in phosphate buffered saline (PBS). A sterile scalpel blade was used to cut a one fourth sized wedge out of each tissue sample for DNA extraction. The biopsy wedges were placed in 2 mL microcentrifuge tubes with 200 μL of PBS and two 4 mm stainless steel grinding balls. Tissues were homogenized with a Retsch MM300 mixer mill (Bio-Rad, Hercules, CA) for 2 min at 30 Hz. DNA was extracted from the homogenate using the DNeasy Blood and Tissue Kit (QIAGEN, Valencia, CA).

Buffy coat samples stored in RNA*later* were centrifuged in 2.0 mL microcentrifuge tubes at 5000 × *g* for 5 min. The supernatant was removed, and the remaining cell pellet was resuspended in 200 μL of PBS with 200 μL of lysis buffer (QIAGEN). DNA was extracted using the DNeasy Blood and Tissue Kit.

Real time PCR was performed on extracted DNA using primers designed to amplify a segment of the *Orientia* 47-kD antigen gene (Additional file 1: Table S1). [13] Of the samples positive for *OT* DNA, nested PCR to amplify a portion of the 56-kD protein gene was performed as previously described (Additional file 1: Table S1). [14] PCR amplicons were cloned using chemically competent *E. coli* cells and the TOPO TA Cloning Kit (Life Technologies). Plasmid DNA was purified with the PureLink Plasmid Miniprep Kit (Life Technologies) and sequenced using a 3130xl Genetic Analyzer (Life Technologies).

The partial 56-kD sequences were trimmed of primer sequences using EditSeq of the Lasergene Core Suite software version 12.0 (DNASTAR, Madison, WI) and then identified by comparing them with sequences available in GenBank via BLAST database search (http,//blast.ncbi.nlm.nih.gov). Phylogenetic analyses of the study and reference sequences were performed using MegAlign of the Lasergene Core Suite (DNASTAR). Sequence alignment was performed using CLUSTAL W, and a phylogenetic tree was constructed with 1,000 bootstrap replications using the neighbor-joining method. For residue conservation analysis, nucleotide sequences were translated from open reading frames of DNA sequences using EditSeq. These nucleotide sequences were then aligned and analyzed using MegAlign.

### Results

During the period of six months from January - August 2013, 19 eschar biopsies and 6 buffy coat samples were collected from study site 1 (Balapitiya), and 1 eschar biopsy and 2 buffy coat samples were collected from study site 2 (Ragama). Real time PCR for the *Orientia* 47-kD protein gene showed amplicons in 16 samples (15/25 at study site 1 and 1/3 at study site 2). Of those positive by real time PCR, nested PCR for the 56-kD gene amplified DNA from 12 eschars and 4 buffy coat samples (Additional file 2: Figure S1).

**Fig. 2:**
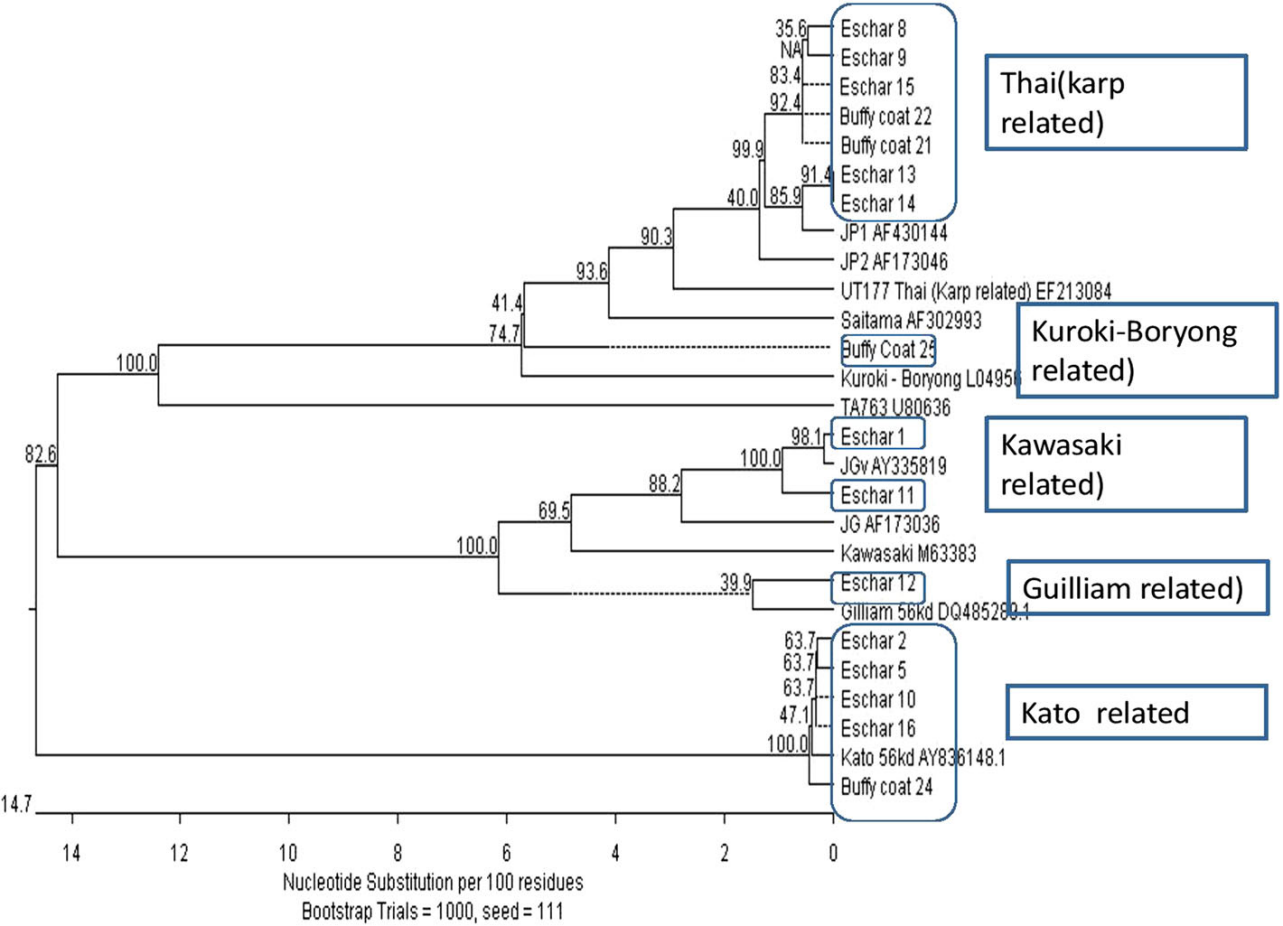
Phylogenetic tree to demonstrate the genetic relationship of *O. tsutsugamushi* isolates from the two study sites

The genotypes of the Location 1 samples showed that seven exhibited close homology with JP1 [distantly related to UT177 Thai (Karp-related)], five had close homology with Kato strain, two had close homology with JGv and JG AF [distantly related to Kawasaki M63383], one had close homology with Gilliam strain. The Location 2 strain was closely related to Kuroki-Boryong L04956 (Fig. 2).

Alignment and residue conservation analysis of the nucleotide sequences demonstrated that genotypic differences between samples conferred nucleotide heterogeneity, which could result in antigenic variation in the studied region (Additional file 3: Figure S2 and Additional file 4: Figure S3).

## Discussion

Originally, three distinctive antigenic prototypes of OT (Karp, Kato, and Gilliam) were described using serologic testing (via complement fixation) [15]. The serologic diversity is based largely on the 56-kDa type specific antigen (TSA), [16]. Later, over 20 additional strains of OT were found [9] by indirect immunofluorescence assays (IFA) using strain- or type-specific monoclonal antibodies or hyperimmune sera which recognize the 56-kDa TSA and by the genetic sequences of this genetically diverse protein. Recent efforts to catalog the strains present in various locations have identified novel subtypes. Multi-locus sequence typing (MLST) analysis of Thai isolates has shown a very high rate of recombination, which along with gene duplication and horizontal gene transfer accounts for the genetic diversity among OT isolates in various locations of Thailand [17, 18].

An increased prevalence of Karp and Karp-like strains has been reported in the Southeast Asian countries, including Malaysia (56%), Thailand (65%), Myanmar (46%), and Philippines (78%) [19–21], Boryong is the predominant strain reported from Korea [22]. However, several studies in India have reported a high prevalence of Kato-like organisms (65%) and the majority have sequence similarity with OT isolates from Korea, Thaiwan, Vietnam and Cambodia [23]. Around 35% were Karp-like, and the rest were related to Kawasaki and Gilliam types.

This is the first study from Sri Lanka to describe the genotypes of OT, in two geographical areas that are about 100 km apart along the western coastal belt within the wet zone of Sri Lanka with probably similar ecological conditions. We were able to recruit 25 patients from study site 1 and 3 patients in study site 2 during the 6 months of the study. However, there were only 15/25 at study site 1 and 1/3 at study site 2 were positive by real time PCR, nested PCR for the 56-kD gene. The patients who were negative by PCR fulfilled criteria for recruitment to the study and had very high IFA-IgG titres against *Orientia* antigens. However, it is very likely that they had either received anti-rickettsial antibiotics prior to admission to hospital by the general practitioners or were recruited at a very late stage of the infection and therefore had already cleared the agent from their blood or tissues.

Although the study included only 16 positive samples, it is interesting to note that we have observed great genetic diversity of OT within the relatively small geographical areas that were studied. In contrast to what has been reported from India, but similar to reports from southeastern parts of Asia, we noted a slightly higher prevalence of Karp [Thai-related] genotypes followed by Kato-related, Kawasaki-related and Gilliam-related genotypes. However, it is interesting to note the close homology with the Kuroki-Boryong organism in one patient from study site 2 (Ragama) who, like the others, had never travelled out of Sri Lanka. The main limitation of this study is that it was carried out over a short period of 6 months and included only 16 positive samples (15 from study site 1 and only 1 from study site 2). Although we observed a close temporal distribution of genotypes within the 6 months, a longer study recruiting more epidemiologically representative samples, is likely to give more insight into the pattern of circulation of the different strains of *O. tsutsugamushi* in the two selected geographical regions. Design of such a study requires an epidemiological survey of the two study sites in order to understand the exact prevalence of this re-emerging infection in the two areas – such data is currently lacking.

A 2011 study in Korea comparing the clinical severity between Boryong and Karp genotypes suggested that eschars and rashes were found in 97% and 94% of the patients infected with the Boryong cluster compared to 73.7% and 68.4% of the patients infected with the Karp cluster, respectively, suggesting clinical variation among infections with different serotypes of OT [24]. In a previous study conducted in the same geographical region, we found a high eschar detection rate (89%) that is in keeping with the above findings [25].

Since this study analyzed only 16 positive samples, it is difficult to arrive at conclusions on the complete composition of OT genotypes in Sri Lanka. However, since Sri Lanka has diverse ecological and environmental conditions within the matter of 2–3 h travel, a larger scale study that covers all ecological zones could contribute to understanding of the complex diversity of OT infection and its transmission within the country.

## Conclusions

We observed five genotypes of *O. tsutsugamushi* demonstrating great genetic diversity in relatively small geographic areas of Sri Lanka, with the majority falling into the Karp-related clade. We believe that such knowledge will contribute to potential prediction of clinical illness, production of future diagnostic tests, and development of vaccines.

## Additional files

Additional file 1:
**Table S1.** Primers designed to amplify a segment of the Orientia 47-kD antigen gene and a portion of the 56-kD protein gene. (DOCX 14 kb)
Additional file 2:
**Figure S1.** Example of representative PCR amplicons on a 1% DNA agarose gel. Lane 1, 100 bp DNA ladder; lane 2, eschar #8 (Karp-related); lane 3, eschar #2 (Kato-related); lane 4, eschar #12 (Gilliam-related); lane 5, negative control; lane 6, Karp strain positive control; lane 7, Kato strain positive control; lane 8, Gilliam strain positive control. (JPG 9 kb)
Additional file 3:
**Figure S2.** Analysis of nucleotide sequence alignment showing the percent identity and divergence as each eschar and buffy coat nucleotide sequence is compared to one another. (JPG 121 kb)
Additional file 4:
**Figure S3.** Graphical report showing matching versus differing residues within the nucleotide sequence alignment generated from eschars and buffy coats. (JPG 210 kb)

## Abbreviations

CA: Canada
DNA: Deoxy ribonucleic acis
IFA: Indirect immunofluorescence antibody
IgG: Immunoglobin G
MLST: Multilocus sequence typing
NY: New York
OT: *Orientia tsutsugamushi*
PBA: Phosphate buffered salina
PCR: Polymerase chain reaction
RNA: Ribonucleic acid
ST: Scrub typhus
TSA: Type specific antigen
USA: United States of America
