# Supplementary material for: Genotypic characterization of Orientia tsutsugamushi from patients in two geographical locations in Sri Lanka
Source: BMC Infect Dis. 2017 Jan 13;17:67. doi: 10.1186/s12879-016-2165-z (PMC5237229; doi:10.1186/s12879-016-2165-z)
Supplement: Additional file 1: — Table S1. Primers designed to amplify a segment of the Orientia 47-kD antigen gene and a portion of the 56-kD protein gene. (DOCX 14 kb) [file 12879_2016_2165_MOESM1_ESM.docx]

| Primers | | Oligonucleotide Sequence |
| --- | --- | --- |
| OtsuFP630 | Real time | 5’-AACTGATTTTATTCAAACTAATGCTGCT-3’ |
| OtsuRP747 | Real time | 5’-TATGCCTGAGTAAGATACRTGAATRGAATT-3’ |
| 34 | Conventional | 5’-TCAAGCTTATTGCTAGTGCAATGTCTGC-3’ |
| 55 | Conventional | 5’-AGGGATCCCTGCTGCTGTGCTTGCTGCG-3’ |
| 10 | Nested | 5’-GATCAAGCTTCCTCAGCCTACTATAATGCC-3’ |
| 11 | Nested | 5’-CTAGGGATCCCGACAGATGCACTATTAGGC-3’ |

Table: Oligonucleotide primer sets used to amplify *O. tsutsugamushi* DNA
