# Supplementary figures and images for: Genotypic characterization of Orientia tsutsugamushi from patients in two geographical locations in Sri Lanka
Source: BMC Infect Dis. 2017 Jan 13;17:67. doi: 10.1186/s12879-016-2165-z (PMC5237229; doi:10.1186/s12879-016-2165-z)

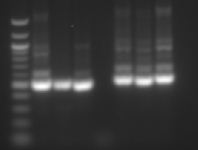

Supplement: Additional file 2: — Figure S1. Example of representative PCR amplicons on a 1% DNA agarose gel. Lane 1, 100 bp DNA ladder; lane 2, eschar #8 (Karp-related); lane 3, eschar #2 (Kato-related); lane 4, eschar #12 (Gilliam-related); lane 5, negative control; lane 6, Karp strain positive control; lane 7, Kato strain positive control; lane 8, Gilliam strain positive control. (JPG 9 kb) [file 12879_2016_2165_MOESM2_ESM.jpg]

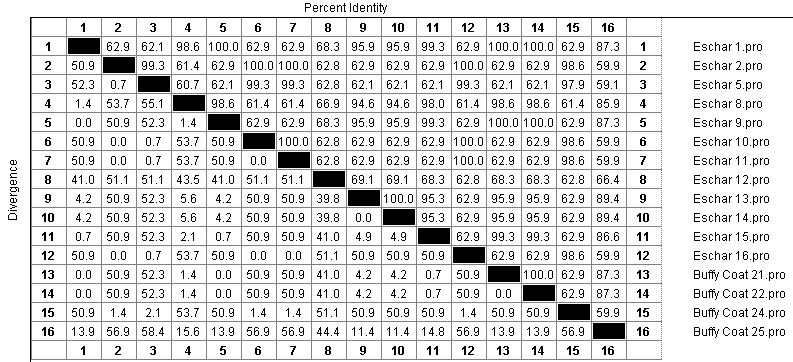

Supplement: Additional file 3: — Figure S2. Analysis of nucleotide sequence alignment showing the percent identity and divergence as each eschar and buffy coat nucleotide sequence is compared to one another. (JPG 121 kb) [file 12879_2016_2165_MOESM3_ESM.jpg]

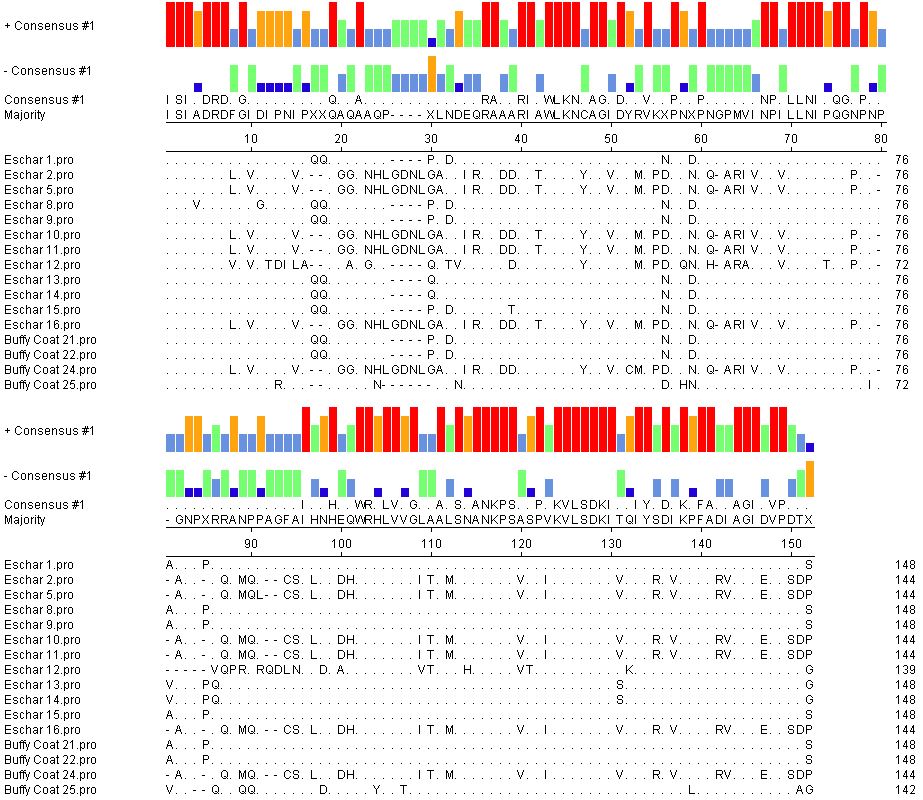

Supplement: Additional file 4: — Figure S3. Graphical report showing matching versus differing residues within the nucleotide sequence alignment generated from eschars and buffy coats. (JPG 210 kb) [file 12879_2016_2165_MOESM4_ESM.jpg]
